# Severe Hemolysis in a COVID-19 Patient with Paroxysmal Nocturnal Hemoglobinuria

**DOI:** 10.1155/2021/6619177

**Published:** 2021-07-13

**Authors:** Steve Biko Otieno, Alaa Altahan, Fnu Kaweeta, Saradasri Karri, Fnu Alnoor, Robert Johnson

**Affiliations:** ^1^The University of Tennessee Health Science Center, Department of Hematology and Oncology, 19 S. Manassas, Memphis, TN 38103, USA; ^2^The Veterans Affairs Medical Center, 1030 Jefferson Ave, Memphis, TN 38104, USA; ^3^The West Cancer and Research Institute, 7945 Wolf River Blvd, Germantown, TN 38138, USA; ^4^The University of Tennessee Health Science Center, Department of Pathology, 930 Madison Ave, Memphis, TN 38103, USA

## Abstract

Severe acute respiratory syndrome coronavirus 2 (SARS-CoV-2), the virus that causes coronavirus disease 2019 (COVID-19), has been demonstrated to be able to activate complement, making patients with deficiency in negative complement regulation, such as paroxysmal nocturnal hemoglobinuria (PNH), particularly vulnerable to complement-mediated cell damage. We report a case of a patient who presented with fatigue, facial swelling, and upper respiratory infection (URI) symptoms and was found to have COVID-19 with laboratory tests showing severe hemolysis and pancytopenia secondary to PNH.

## 1. Background

Severe acute respiratory syndrome coronavirus 2 (SARS-CoV-2) has infected more than 150 million worldwide and caused over 3 million deaths since December 2019, and these numbers continue to rise. It was declared a pandemic by the World Health Organization (WHO) on March 11, 2020. SARS-CoV-2 has been shown to be able to activate complement, making patients with a deficiency in negative complement regulation, such as paroxysmal nocturnal hemoglobinuria (PNH), particularly vulnerable to complement-mediated cell damage [1, 2] Patients with PNH have a mutation in the *PIG-A* gene which controls a key step in the biosynthesis of cell surface linker glycosyl-phosphatidyl-inositol (GPI) which tethers the negative complement regulators CD55 and CD59 to cell surfaces. The lack of the GPI linker results in the absence of these two key negative complement regulators [3]. On erythrocytes, the absence of the GPI linker results in complement-mediated hemolysis which is a common presentation of PNH. We report a case of a patient with COVID-19 who was diagnosed with PNH after she presented with fatigue, facial swelling, and URI symptoms with laboratories showing severe hemolysis and pancytopenia.

## 2. Case Presentation

The patient is a 19-year-old female with no significant past medical history who presented to the Emergency Department (ED) with left-sided facial swelling. 3 weeks prior to presentation, she developed cough, subjective fever, severe fatigue, and swelling of the gums of her left teeth. The gum swelling got worse, and she started having facial swelling. She underwent a COVID-19 test in the community prior to presentation but had not received the results at the time of presentation. The jaw swelling worsened and started draining pus despite taking amoxicillin prescribed by her dentist. She presented to the ED for further evaluation. On review of systems, she denied any history of recurrent infections, anemia, bleeding, rashes, jaundice, or thrombosis. Her only medication was amoxicillin. She denied smoking, alcohol, or illicit drug use. Family history was notable for a cousin, aunt, and grandmother with leukemia.

On exam, her temperature was 37.3°C, blood pressure was 114/75, heart rate was 130, and oxygen saturation was 99% on room air. She was noted to have left face and neck swelling. Lungs were clear to auscultation bilaterally. She had no bruises, petechiae, or lymphadenopathy. The rest of her exam was unremarkable. A maxillofacial computed tomographic scan (CT) showed diffuse soft tissue edema throughout the left face without drainable fluid collection to suggest an abscess. Initial labs showed pancytopenia with a white blood cell count of 2.6 × 10^3^ per microliter (reference range 4.2 × 10^3^ to 10.2 × 10^3^), an absolute neutrophil count of 1.8 × 10^3^ per microliter (reference range 1.8 × 10^3^ to 7.1 × 10^3^), a hemoglobin of 2.7 grams per deciliter (reference range 11.5 to 14.8), and a platelet count of 30 × 10^3^ per microliter (reference range 150 × 10^3^ to 400 × 10^3^). Other notable laboratory results were a total bilirubin level of 1.3 milligrams per deciliter (reference range 0.2 to 1.0), haptoglobin of 22 milligrams per deciliter (reference range 30–200), lactate dehydrogenase (LDH) of 2218 U per liter (reference range 84 to 246), reticulocyte count of 55.3 × 10^3^ per microliter (reference range 30–90), prothrombin time of 20.3 seconds (reference range 23.2 to 34.1), international normalized ratio of 1.3 (reference range 0.8 to 1), partial thromboplastin time of 15.5 seconds (reference range 11.7 to 14.5), and fibrinogen of 514 milligrams per deciliter (reference range 208 to 475). A direct Coombs test was negative. Parvovirus B19 IgG was positive with a titer of 7.4 (reference range <0.9), but the IgM was negative. The results of the rest of her initial tests are shown in Table 1. She received four units of packed red blood cells (pRBCs) and was started on broad-spectrum antibiotics.

The patient developed a fever with a maximum temperature of 38.5°C after admission. Her COVID-19 test result returned as positive. Since the initial labs were consistent with hemolysis in the context of pancytopenia, a peripheral smear was ordered as well as flow cytometry on peripheral blood and flow cytometry for PNH. A bone marrow aspiration and core biopsy were performed. A CT chest, abdomen, and pelvis did not reveal any adenopathy and was negative for pulmonary embolism.

**Figure 1:**
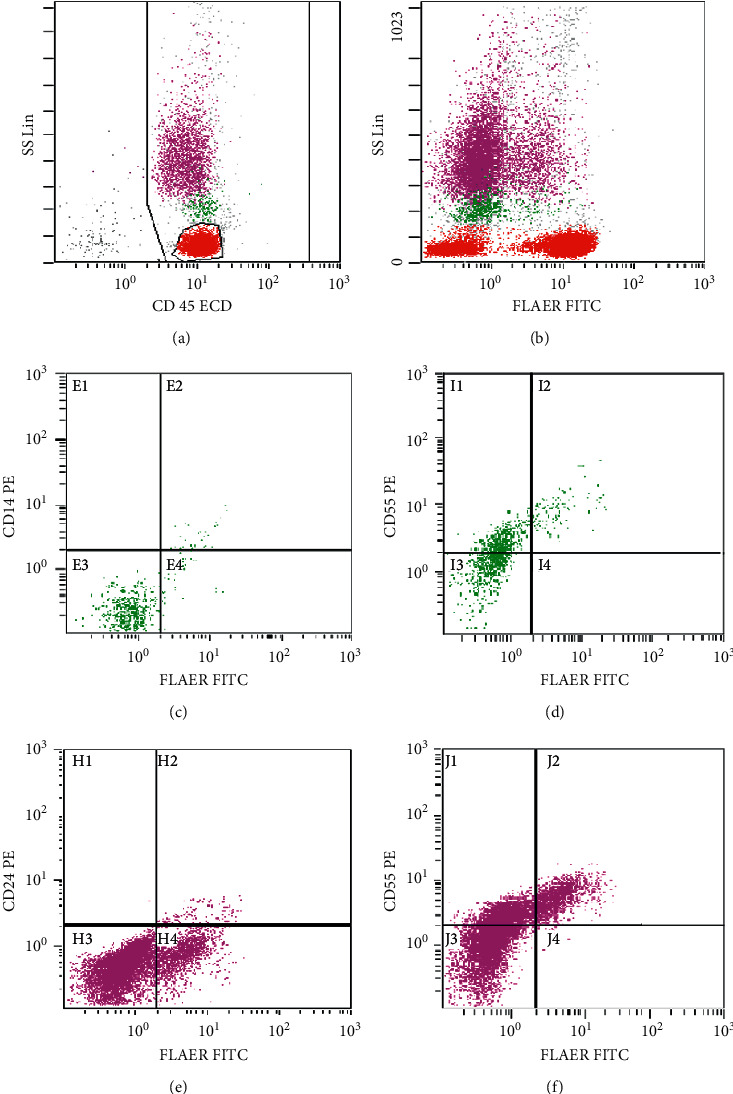
PNH flow cytometry. Lymphocytes (red), monocytes (green), and granulocytes (pink) were displayed on the CD 45 side scattered (SS) plot (a). The SS plot demonstrating fluorescein-labeled-proaerolysin- (FLAER-) negative different cell populations (b). 51% of monocytes and 52% of granulocytes had PNH immunophenotype; FLAER-/CD14-/CD55 and FLAER-/CD24-/CD55, respectively ((c)–(f)).

**Figure 2:**
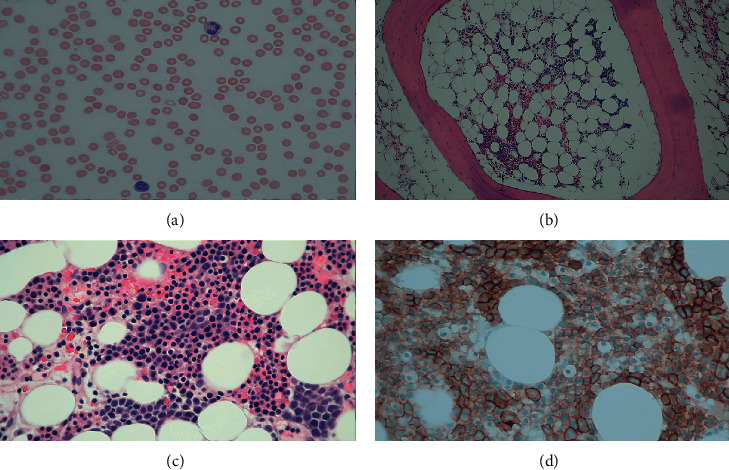
Peripheral blood and bone marrow biopsy. The peripheral blood smear showed leukopenia with absolute neutropenia and thrombocytopenia. The observed neutrophils and lymphocytes were mature without significant morphological abnormality. Schistocytes were not present (a). Bone marrow biopsy showed large areas of the marrow that are less than 10% cellular (b); however, focally, the marrow is 40–50% cellular with erythroid predominant trilineage hematopoiesis (c). Immunohistochemical stains performed with appropriate controls demonstrated numerous E-cadherin-positive erythroid precursors (d).

Flow cytometry was positive for PNH (Figure 1). The peripheral blood smear showed leukopenia with absolute neutropenia and thrombocytopenia. The observed neutrophils and lymphocytes were mature without significant morphological abnormality. Schistocytes were not present (Figure 2(a)). Bone marrow biopsy showed large areas of the marrow that were less than 10% cellular (Figure 2(b)); however, focally, the marrow is 40–50% cellular with erythroid predominant trilineage hematopoiesis (Figure 2(c)). Megakaryocytes were present but markedly decreased without any significant dysplasia. Few mature neutrophils were present. Blasts were not increased. Immunohistochemical stains performed with appropriate controls demonstrated numerous E-cadherin-positive erythroid precursors (Figure 2(d)). There were rare CD34-positive blasts and few scattered CD3-positive T cells and CD20-positive B cells. Terminal deoxynucleotidyl transferase (TdT) stain was negative. Bone marrow aspirate showed small hypocellular spicules with erythroid predominance. Some of these erythroid precursors were binucleated and had nuclear budding or irregular nuclear contours. Neutrophil precursors were present but infrequent. Blasts were not increased. A cytogenetics study revealed normal female karyotype (46XX). No mutations were detected by targeted next-generation sequencing.

The patient was treated with 2 doses of 600 milligrams of the C5 complement inhibitor eculizumab as inpatient with improvement in counts. She was seen in clinic after discharge, and she received 2700 mg of the longer acting C5 complement inhibitor ravulizumab a week after the last dose of eculizumab and a 3300 mg dose 2 weeks later. Her last blood counts showed a white blood cell count of 4 × 10^3^ per microliter, absolute neutrophil count of 2.2 × 10^3^ per microliter, hemoglobin level of 7.8 grams deciliter, and platelet count of 50 × 10^3^ per microliter. Haptoglobin was 30, and LDH was normal. In the subsequent visits, the patient's pancytopenia persisted, and she was referred to a transplant center for evaluation.

## 3. Discussion

**Figure 3:**
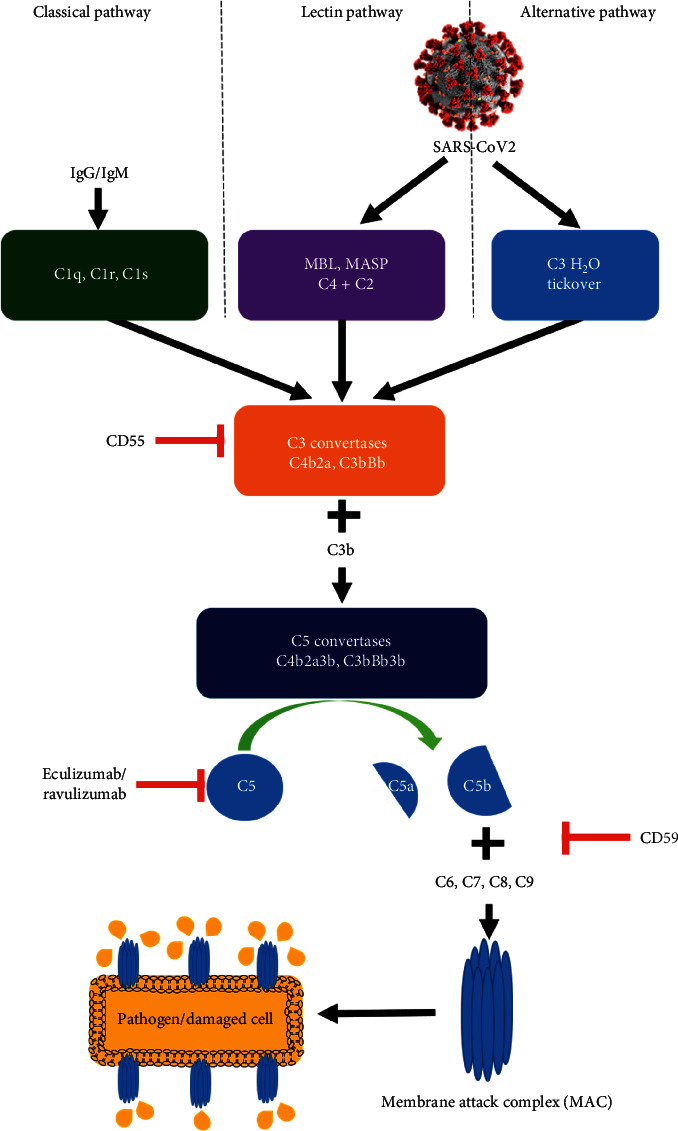
The complement system and its inhibitors. SARS-CoV-2 activates complement through the lectin and alternative pathways. CD55 negatively regulates the activity of C3 convertase and CD59 negatively regulates the formation of the membrane attack complex. Eculizumab and ravulizumab prevent the cleavage of C5 and prevent formation of the membrane attack complex. MBL: mannose-binding lectin; MASP: MBL-associated serine protease.

The patient in this case presented with a tooth infection, severe fatigue, and upper respiratory infection symptoms with work-up revealing severe hemolysis in the setting of COVID-19. Flow cytometry analysis of peripheral blood revealed a PNH clone. PNH is characterized by severe fatigue, smooth muscle dystonias, hemolysis, and thrombosis [3]. The patient did not have a history of hemolysis or thrombosis. Iron studies were not consistent with a chronic blood loss process as the patient had a normal mean corpuscular volume (MCV). The underlying pathophysiology of PNH is the absence of the negative complement regulatory proteins CD55 and CD59 on the surface of erythrocytes (Figure 3). Processes that activate the complement pathway make these erythrocytes vulnerable to complement-mediated hemolysis. SARS-CoV-2 has been implicated in overactivation of the complement system. Gao et al. reported that SARS-CoV-2 can activate the lectin complement pathway [1]. In another study, Yu et al. demonstrated that the SARS-CoV-2 spike proteins can activate the alternative complement pathway and also showed that disrupting interaction of the spike proteins and factor *D* or C5 inhibited this immunopathology [2]. The COVID-19 of this patient activated complement, resulting in the diagnosis of PNH in this previously asymptomatic patient. A complement-mediated thrombotic microangiopathy (TMA) has also been described in COVID-19 [4]. This type of TMA is usually characterized by hemolytic anemia, thrombocytopenia, and end-organ damage [4]. While the patient in this case had hemolytic anemia and thrombocytopenia, she did not have any end-organ damage suggesting that her pathophysiology was not driven by a TMA. In addition, TMAs usually have schistocytes on the peripheral blood smear. No schistocytes were observed on the peripheral blood smear for the patient in this case report.

PNH is classified into 3 groups: (1) subclinical PNH which usually has few PNH clones but no clinical or laboratory manifestation of hemolysis or thrombosis; (2) classical PNH, which has clinical and laboratory manifestations of hemolysis and thrombosis; and (3) PNH in the setting of other marrow failure syndromes such as aplastic anemia or myelodysplastic syndrome [5]. It is important to note that a bone marrow biopsy in cases on PNH typically reveals a normal to increased cellularity with erythroid predominance [3]. The patient in this case had a hypocellular marrow suggesting a secondary process. She had likely had a concomitant severe aplastic anemia. Since the predominant clinical presentation of this patient was complement-mediated hemolysis, the goal of primary treatment was to control the hemolysis through complement inhibition.

The use of complement inhibitors such as eculizumab or ravulizumab is well established in treating PNH. Both drugs are monoclonal antibodies against the C5 component of complement preventing its cleavage into to C5a and C5b. This prevents the terminal complement cascade and the formation of the membrane attack complex (MAC), as shown in Figure 3. Pike et al. reported an institutional experience of 4 patients with COVID-19 who had a history of PNH and were on complement inhibitors [6]. Kulasekararaj et al. also reported an institutional experience of another 4 patients with COVID-19 and a history of PNH [7]. Two of these patients were treatment naïve, and two were established on complement inhibitor therapy [7]. The results of a small study of 80 patients suggested that eculizumab may improve survival and reduce hypoxia in patients with severe COVID-19 [8]. However, there is no approved indication yet for use in COVID-19 patients. Clinical trials investigating the use of eculizumab (https://clinicaltrials.gov/ct2/show/NCT04288713 and https://clinicaltrials.gov/ct2/show/NCT04346797) and ravulizumab (https://clinicaltrials.gov/ct2/show/NCT04570397, https://clinicaltrials.gov/ct2/show/NCT04390464, and https://clinicaltrials.gov/ct2/show/NCT04369469) in COVID-19 are ongoing. Other potential therapeutic agents targeting the complement pathway include narsoplimab which is a fully human Ig4 monoclonal antibody directed against the mannan-binding lectin-associated serine protease-2 (MASP-2), an effector enzyme of the lectin pathway of the complement system [9], AMY-101, a peptide inhibitor of the C3 complement [10], and Zilucoplan, a C5 complement inhibitor [11].

## 4. Conclusions

This 19-year-old female with undiagnosed PNH became symptomatic in the context of a SARS-COV-2 infection. Management of PNH in the setting of SARS-CoV-2 infection poses a great challenge. SARS-CoV-2 has been shown to activate complement, and patients with PNH lack key inhibitors of complement regulation, CD55 and CD59, and are, therefore, predisposed to complement-mediated damage. This case raises a great concern for patients who, at baseline, have clinically evident PNH. In such patients, a SARS-CoV-2 infection would potentially cause significant morbidity and mortality. In this patient population, it may be prudent to consider anticomplement therapy, for patients not on this therapy, to mitigate potentially catastrophic SARS-CoV-2-mediated complement activation. This case shows that complement inhibitors may potentially be effective in controlling SARS-CoV-2-mediated complement-activation-dependent hemolysis in PNH patients.

## Figures and Tables

**Table 1:** Laboratory values.

	Reference ranges	Initial labs	Before starting eculizumab	After 2 doses of eculizumab
White cells (thou/mcL)	4.2–10.20	2.6	3.2	5.4
Hemoglobin (g/dL)	11.5–14.8	2.7	9.0	8.2
Hematocrit (%)	34.6–43.8	8.5	26.6	24.9
Platelets (thou/mcL)	150–400	30	14	45
Absolute neutrophil count (thou/mcL)	1.8–7.1	1.8	0.2	3.0
Retic count (%)	0.5–2.0	2.57	1.73	5.63
Iron (mcg/dL)	50–170	58	152	
Ferritin (ng/mL)	8.0–252.0	246		517
Transferrin (mg/dL)	200–360	250	176	
Vitamin B12 (pg/mL)	211–911	345	552	
Lactate dehydrogenase (unit/L)	84–246	2218	566	189
Haptoglobin (mg/dL)	30–200	22	27	13
Fibrinogen (mg/dL)	208–475	514	391	
Partial thrombin time (sec)	11.7–14.5	20.3	24.1	29.4
International normalized ratio	0.8–1.0	1.3	1.0	1.0
Direct antiglobulin test	Negative	Negative	Negative	
Sodium (mmol/L)	136–145	139	141	140
Potassium (mmol/L)	3.5–5.1	3.6	3.5	3.8
Chloride (mmol/L)	98–107	108	106	109
Carbon dioxide (mmol/L)	22–32	19	29	24
Urea nitrogen (mg/dL)	7–18	19	14	5
Creatinine (mg/dL)	0.52–1.21	1.15	0.43	0.58
Alkaline phosphatase (unit/L)	45–117	82	92	197
Aspartate aminotransferase (unit/L)	15–37	75	26	22
Alanine aminotransferase (unit/L)	13–56	28	76	21
Total bilirubin (mg/dL)	0.2–1.0	1.3	0.7	0.6

## Data Availability

All data analyzed during this study are included in this published article.
